# Data concerning adsorption equilibria of carbon dioxide, nitrogen and oxygen over a zeolite molecular sieve 13X for the modelling of carbon dioxide capture from gaseous mixtures by adsorptive processes

**DOI:** 10.1016/j.dib.2020.105638

**Published:** 2020-04-30

**Authors:** Manfred Jaschik, Marek Tanczyk, Jolanta Jaschik, Aleksandra Janusz-Cygan

**Affiliations:** Institute of Chemical Engineering, Polish Academy of Sciences, ul. Baltycka 5, 44-100 Gliwice, Poland

**Keywords:** adsorption equilibria, zeolite 13X, gravimetric method, vacuum swing adsorption, CO_2_ capture

## Abstract

Experimental adsorption isotherms of carbon dioxide, nitrogen and oxygen at 293, 313 and 333 K over a zeolite molecular sieve 13X Grace are presented. The data were used in the simulations of the hybrid VSA-membrane process for carbon dioxide capture from flue gas as presented in a related article entitled “The performance of a hybrid VSA-membrane process for the capture of CO_2_ from flue gas” [1]. A representative sample of ZSM 13X Grace (149.7 mg) was prepared using the Microscal Spinning Riffler. Adsorption equilibria were determined by a gravimetric method, which uses a microbalance IGA003, Hiden Isochema Ltd., UK at temperatures of 293, 313 and 333 K. Every adsorption isotherm was started at 0 bar. For CO_2_ the equilibrium concentration reaches 3.755-4.857 mol kg^−1^ at the maximum pressure of 1 bar. In the case of N_2_ and O_2_ the equilibrium concentration reaches, respectively, 0.721-1.255 mol kg^−1^ and 0.299-0.531 mol kg^−1^ at the maximum pressure of 5 bar. Data may be reused in any adsorptive CO_2_/N_2_/O_2_ separation process which uses ZMS 13X as an adsorbent.

**Specifications Table**

**Table:** 

**Subject**	Filtration and Separation
**Specific subject area**	Gas separation by adsorption
**Type of data**	Table Chart
**How data were acquired**	Adsorption equilibrium of pure gases was measured gravimetrically, using the microbalance IGA003, Hiden Isochema Ltd., UK. For a pure single gas the relative sample mass changes in the equilibrium state were determined at a given temperature and pressure step, which took into account the buoyancy correction as calculated with the device's software. Experimental parameters were programmed, controlled and recorded by the software provided by Hiden Isochema Ltd.
**Data format**	Raw Analyzed
**Parameters for data collection**	Sample preparation: degassing overnight at 593 K Pressure range: 0-1 bar for CO_2_ and 0-5 bar for N_2_ and O_2_ Temperatures: 293, 313 and 333 K
**Description of data collection**	Mass adsorbed was recorded after reaching equilibrium at a given temperature and pressure. Raw data are given in the supplementary material.
**Data source location**	Institute of Chemical Engineering, Polish Academy of Sciences Gliwice Poland
**Data accessibility**	With the article in the supplementary material.
**Related research article**	M. Jaschik, M. Tanczyk, J. Jaschik, A. Janusz-Cygan, The performance of a hybrid VSA-membrane process for the capture of CO_2_ from flue gas, International Journal of Greenhouse Gas Control, 10.1016/j.ijggc.2020.103037

**Value of the Data**

• The data are necessary for the modelling, simulations and design of the capture of CO_2_ by vacuum/pressure swing adsorption which uses ZSM 13X as an adsorbent.
• The data may be used by a broad scientific community in the adsorbent selection, modelling and design of gas separation processes.
• Comparison with data acquired by other methods may be done as well as further experimental studies on multicomponent adsorption equilibria.

## 1 Data

**Fig. 1:**
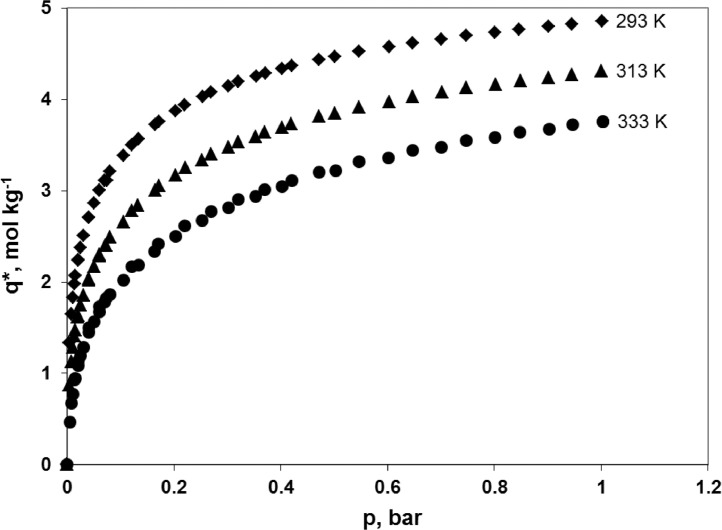
Adsorption isotherms of pure CO_2_ on ZMS 13X Grace, determined gravimetrically at temperature of 293 K, 313 K and 333 K, and pressure of 0-1 bar using the microbalance IGA003, Hiden Isochema Ltd., UK.

**Fig. 2:**
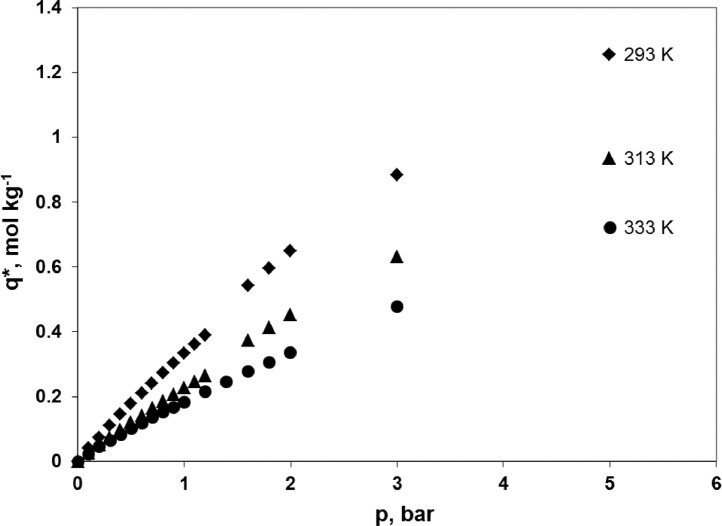
Adsorption isotherms of pure N_2_ on ZMS 13X Grace, determined gravimetrically at temperature of 293 K, 313 K and 333 K, and pressure of 0-5 bar using the microbalance IGA003, Hiden Isochema Ltd., UK.

**Fig. 3:**
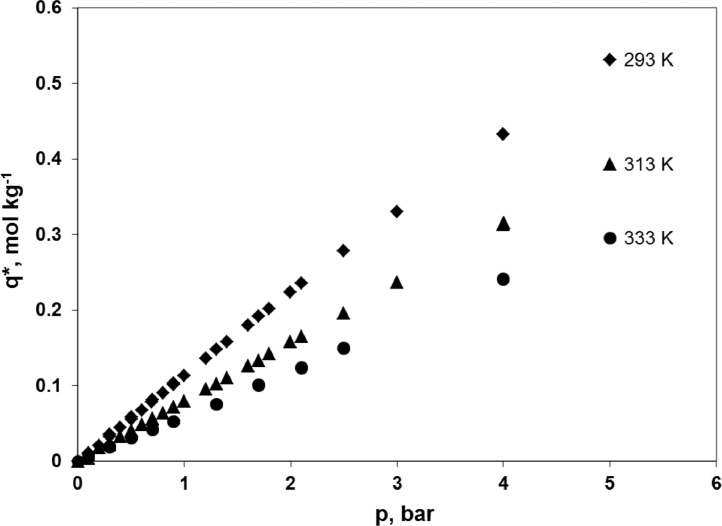
Adsorption isotherms of pure O_2_ on ZMS 13X Grace, determined gravimetrically at temperature of 293 K, 313 K and 333 K, and pressure of 0-5 bar using the microbalance IGA003, Hiden Isochema Ltd., UK.

Experimental points concerning the adsorption equilibria of carbon dioxide on ZMS 13X Grace at 293, 313 and 333 K are presented in Fig. 1 and the raw data are given in Table 1. Adsorption points (i.e. determined while the pressure is increased) and desorption points (i.e. determined while the pressure is decreased) are shown together. Experimental points concerning the adsorption equilibria of nitrogen on ZMS 13X Grace at 293, 313 and 333 K are presented in Fig. 2 and the raw data are given in Table 2. Experimental points concerning the adsorption equilibria of oxygen on ZMS 13X Grace at 293, 313 and 333 K are presented in Fig. 3 and the raw data are given in Table 3. Adsorption points and desorption points are shown together.

**Table 1:** Adsorption equilibria of pure CO_2_ on ZMS 13X Grace, determined gravimetrically at temperature of 293 K, 313 K and 333 K, and pressure of 0-1 bar using the microbalance IGA003, Hiden Isochema Ltd., UK.

293 K		313 K		333 K	
p, bar	q*, mol kg^−1^	p, bar	q*, mol kg^−1^	p, bar	q*, mol kg^−1^
0	0	0	0	0	0
0.004	1.338	0.004	0.875	0.005	0.469
0.007	1.652	0.007	1.126	0.007	0.674
0.010	1.833	0.010	1.287	0.010	0.770
0.013	1.979	0.013	1.409	0.013	0.927
0.015	2.074	0.015	1.472	0.015	0.945
0.020	2.237	0.020	1.616	0.020	1.083
0.020	2.243	0.020	1.635	0.020	1.134
0.025	2.380	0.025	1.751	0.025	1.191
0.030	2.511	0.030	1.855	0.030	1.287
0.040	2.707	0.040	2.021	0.040	1.496
0.041	2.711	0.040	2.038	0.040	1.445
0.050	2.862	0.051	2.174	0.050	1.568
0.060	3.001	0.060	2.289	0.060	1.676
0.061	3.003	0.060	2.303	0.060	1.734
0.070	3.116	0.071	2.402	0.071	1.780
0.074	3.115	0.073	2.405	0.073	1.824
0.080	3.213	0.080	2.491	0.080	1.864
0.106	3.386	0.106	2.660	0.106	2.019
0.121	3.497	0.121	2.784	0.121	2.172
0.134	3.570	0.133	2.838	0.134	2.188
0.164	3.722	0.164	3.002	0.164	2.338
0.171	3.758	0.171	3.051	0.171	2.422
0.202	3.873	0.203	3.167	0.203	2.504
0.220	3.940	0.221	3.250	0.220	2.614
0.253	4.030	0.252	3.335	0.253	2.675
0.269	4.079	0.270	3.403	0.269	2.770
0.302	4.149	0.302	3.473	0.302	2.816
0.320	4.193	0.321	3.533	0.320	2.906
0.353	4.250	0.353	3.591	0.352	2.938
0.371	4.287	0.370	3.639	0.369	3.016
0.403	4.333	0.402	3.687	0.402	3.043
0.420	4.367	0.419	3.730	0.420	3.116
0.472	4.437	0.471	3.813	0.471	3.205
0.501	4.467	0.502	3.846	0.502	3.216
0.546	4.523	0.546	3.916	0.546	3.316
0.603	4.575	0.602	3.973	0.601	3.358
0.646	4.620	0.647	4.031	0.647	3.445
0.701	4.661	0.702	4.076	0.701	3.478
0.747	4.701	0.747	4.126	0.747	3.550
0.801	4.735	0.802	4.165	0.801	3.584
0.846	4.769	0.848	4.206	0.847	3.640
0.901	4.800	0.901	4.240	0.903	3.678
0.945	4.827	0.945	4.273	0.946	3.721
1.001	4.857	1.000	4.307	1.003	3.755

**Table 2:** Adsorption equilibria of pure N_2_ on ZMS 13X Grace, determined gravimetrically at temperature of 293 K, 313 K and 333 K, and pressure of 0-5 bar using the microbalance IGA003, Hiden Isochema Ltd., UK.

293 K		313 K		333 K	
p, bar	q*, mol kg^−1^	p, bar	q*, mol kg^−1^	p, bar	q*, mol kg^−1^
0	0	0	1.07E-06	0	0
0.102	0.041	0.098	0.024	0.102	0.024
0.199	0.075	0.199	0.050	0.198	0.046
0.297	0.110	0.298	0.073	0.302	0.064
0.400	0.145	0.399	0.097	0.402	0.083
0.498	0.178	0.500	0.120	0.503	0.101
0.600	0.211	0.600	0.142	0.603	0.119
0.698	0.242	0.701	0.163	0.703	0.136
0.799	0.273	0.801	0.185	0.803	0.152
0.900	0.303	0.900	0.205	0.898	0.167
1.002	0.333	1.002	0.226	1.001	0.184
1.099	0.361	1.099	0.245	1.199	0.215
1.199	0.389	1.198	0.265	1.398	0.246
1.598	0.543	1.598	0.374	1.598	0.278
1.797	0.597	1.797	0.414	1.797	0.306
1.998	0.650	1.998	0.453	1.998	0.337
2.998	0.884	2.998	0.631	2.998	0.478
4.998	1.255	4.998	0.935	4.999	0.721

**Table 3:** Adsorption equilibria of pure O_2_ on ZMS 13X Grace, determined gravimetrically at temperature of 293 K, 313 K and 333 K, and pressure of 0-5 bar using the microbalance IGA003, Hiden Isochema Ltd., UK.

293 K		313 K		333 K	
p, bar	q*, mol kg^−1^	p, bar	q*, mol kg^−1^	p, bar	q*, mol kg^−1^
0	0	0	0	0	0
0.099	0.009	0.099	0.009	0.100	0.005
0.099	0.011	0.100	0.004	0.100	0.007
0.199	0.021	0.201	0.0175	0.300	0.020
0.300	0.033	0.300	0.025	0.301	0.019
0.300	0.035	0.300	0.022	0.500	0.031
0.399	0.045	0.399	0.033	0.501	0.031
0.500	0.056	0.500	0.041	0.698	0.042
0.500	0.058	0.500	0.039	0.700	0.043
0.599	0.067	0.599	0.049	0.899	0.052
0.697	0.079	0.699	0.057	0.901	0.053
0.698	0.081	0.701	0.055	1.299	0.075
0.800	0.090	0.799	0.064	1.299	0.076
0.899	0.102	0.899	0.072	1.699	0.100
0.901	0.104	0.901	0.072	1.701	0.101
0.998	0.113	0.998	0.080	2.099	0.124
1.200	0.136	1.200	0.095	2.099	0.125
1.299	0.148	1.299	0.103	2.499	0.149
1.400	0.158	1.400	0.111	2.499	0.150
1.600	0.180	1.600	0.126	3.999	0.240
1.699	0.192	1.699	0.133	3.999	0.241
1.798	0.202	1.798	0.142	5.000	0.295
1.999	0.224	1.999	0.158	5.001	0.299
2.099	0.236	2.099	0.165		
2.499	0.278	2.499	0.196		
2.998	0.330	2.998	0.237		
3.998	0.432	3.999	0.313		
3.999	0.432	4.001	0.315		
5.001	0.531	5.001	0.393		
5.001	0.531	5.001	0.393		

## 2 Experimental Design, Materials, and Methods

### 2.1 Materials

Zeolite 13X Grace (SYLOBEAD MS 544, GRACE Davison) in a form of beads (diameter of ∼2 mm) was used. Physicochemical and surface properties were given by adsorbent provider and are as follows. Pore size: ∼8 Å, surface area: 800 m^2^ g^−1^, effective pore volume: 0.25-0.3 cm^3^ g^−1^, thermal conductivity: 0.12 W m^−1^ K^−1^, heat capacity: 0.96 kJ K^−1^ kg^−1^. Before the measurements a representative sample (149.7 mg) of the adsorbent was prepared using the Microscal Spinning Riffler. Pure gases were used in the measurements. Carbon dioxide of at least 99.995% purity was supplied by Messer, while nitrogen, oxygen and helium of at least 99.9999% purity were supplied by Air Products. Gases were used with no further purification.

### 2.2 Methods

Experimental adsorption isotherms for single, pure gases: N_2_, CO_2_ and O_2_ over a zeolite molecular sieve 13X Grace were determined based on gravimetric measurements. The microbalance (IGA003, Hiden Isochema Ltd., UK) with a resolution of 0.2 μg and the buoyancy force correction was used. The IGA gravimetric analyzer is a fully computerized microbalance which allows the adsorption-desorption isotherms and the corresponding kinetics of adsorption or desorption at each pressure step to be determined with the approach to equilibrium being monitored in real time using a computer algorithm. The microbalance is fully thermostated to eliminate the effect of ambient temperature (the temperature variations in the system do not exceed ± 0.2 K). The pressure in the device is set and maintained at the set point by active computer control of inlet/outlet valves throughout the duration of the equilibrium experiments.

IGA microbalance can operate in the pressure range of ∼0 to 2.0 MPa using two precalibrated pressure controllers and in the temperature range from 283.15 to 723.15.15 K using a Huber Ministat CC refrigerated recirculating water bath and a Instron TF50/3/12/F furnace. A Pt100 probe with a resolution of 0.01 K is located inside the reactor near the sample container. The experiments performed using the gravimetric analyzer IGA are programmed, controlled and recorded by the software provided by Hiden Isochema Ltd. The buoyancy correction is calculated with this software and taken into account while determining a relative sample mass change in equilibrium.

The container with the representative sample was placed in the tubular reactor. The measurements were conducted in the static mode, i.e. the sample reactor was filled with a gas until a desired pressure was reached, and then the gas supply was cut off until the equilibrium was established.

Before starting the measurements the sample was degassed overnight at 593 K under vacuum using a Vacuubrand GMH-MD1 vacuum pump and an Pfeiffer TMU 017 P turbomolecular pump which achieves an ultrahigh vacuum of 1 × 10^−6^ Pa. Then, a sample density (2.38 g cm^−3^) was determined by measuring a helium displacement isotherm at 293 K.

Isotherms were determined starting from oxygen (the least absorbable gas) and ending on carbon dioxide. An intermediate short outgassing steps were used while changing a gas. For a given gas the measurements were started from the highest (323 K) to the lowest temperature (293 K). While determining the isotherm for a given temperature, the pressure was changed step by step, beginning from the vacuum. For each incremental pressure change the dynamic uptake curve (i.e. sample mass change with time) was recorded until the equilibrium was reached for a given pressure. The time necessary to determine a single isotherm point was set at 40 min and all this time the pressure was maintained at a given constant level.

Raw data recorded within the IGA's software and derived from it include pressure, temperature and a relative mass change of the sample (mass adsorbed) at equilibrium state. These data are presented in the supplementary material.

## Declaration of Competing Interest

The authors declare that they have no known competing financial interests or personal relationships which have, or could be perceived to have, influenced the work reported in this article.

## Appendix Supplementary materials

Image, application 1
